# Supplementary material for: Towards unified AI-driven fracture mechanics: the extended deep energy method (XDEM)
Source: Nat Commun. 2026 Aug 15;17:8492. doi: 10.1038/s41467-026-76748-1 (PMC13484471; doi:10.1038/s41467-026-76748-1)
Supplement: Supplementary file 3 [file 41467_2026_76748_MOESM3_ESM.zip › pic/Results/Stress_intensity_factor/Mixed/beta_15/crack_u1_beta15.pdf]

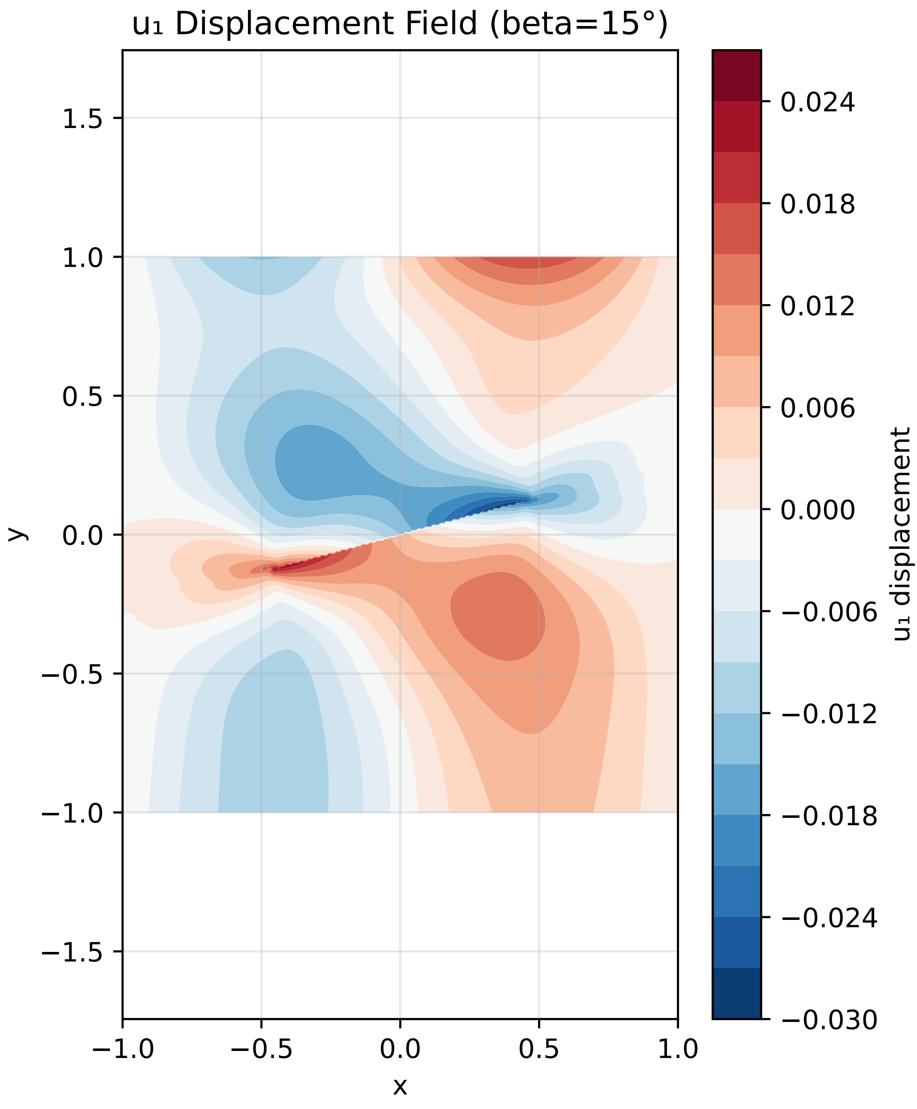

u₁ Displacement Field (beta=15°)
1.5
1.0
0.5
0.0
−0.5
−1.0
−1.5
y
−1.0
−0.5
0.0
0.5
1.0
x
0.024
0.018
0.012
0.006
0.000
−0.006
−0.012
−0.018
−0.024
−0.030
u₁ displacement
